# Nutrient Induced Type 2 and Chemical Induced Type 1 Experimental Diabetes Differently Modulate Gastric GLP-1 Receptor Expression

**DOI:** 10.1155/2015/561353

**Published:** 2015-03-29

**Authors:** Olga Bloch, Efrat Broide, Gilad Ben-Yehudah, Dror Cantrell, Haim Shirin, Micha J. Rapoport

**Affiliations:** ^1^Diabetes and Immunology Research Laboratory, Assaf Harofeh Medical Center Affiliated to the Sackler Faculty of Medicine, Tel-Aviv University, 70300 Zerifin, Israel; ^2^Institute of Gastroenterology, Assaf Harofeh Medical Center Affiliated to the Sackler Faculty of Medicine, Tel-Aviv University, 70300 Zerifin, Israel; ^3^Department of Internal Medicine “C”, Assaf Harofeh Medical Center Affiliated to the Sackler Faculty of Medicine, Tel-Aviv University, 70300 Zerifin, Israel

## Abstract

T2DM patients demonstrate reduced GLP-1 receptor (GLP-1R) expression in their gastric glands. Whether induced T2DM and T1DM differently affect the gastric GLP-1R expression is not known. This study assessed extrapancreatic GLP-1R system in glandular stomach of rodents with different types of experimental diabetes. T2DM and T1DM were induced in *Psammomys obesus* (PO) by high-energy (HE) diet and by streptozotocin (STZ) in Sprague Dawly (SD) rats, respectively. GLP-1R expression was determined in glandular stomach by RT PCR and immunohistomorphological analysis. The mRNA expression and cellular association of the GLP-1R in principal glands were similar in control PO and SD rats. However, nutrient and chemical induced diabetes resulted in opposite alterations of glandular GLP-1R expression. Diabetic PO demonstrated increased GLP-1R mRNA expression, intensity of cellular GLP-1R immunostaining, and frequency of GLP-1R positive cells in the neck area of principal glands compared with controls. In contrast, SD diabetic rats demonstrated decreased GLP-1 mRNA, cellular GLP-1R immunoreactivity, and frequency of GLP-1R immunoreactive cells in the neck area compared with controls. In conclusion, nutrient and chemical induced experimental diabetes result in distinct opposite alterations of GLP-1R expression in glandular stomach. These results suggest that induced T1DM and T2DM may differently modulate GLP-1R system in enteropancreatic axis.

## 1. Introduction

GLP-1 replacement therapy is commonly advocated in T2DM due to its major pancreatic insulinotropic and glucagon suppression effects [1]. However, the incretin hormone GLP-1 regulation of glucose homeostasis is mediated also by multiple extrapancreatic effects at various GLP-1R positive target organs including the stomach. GLP-1 is known as a potent inhibitor of several gastrointestinal functions such as gastric acid secretion, gastric emptying, and gastrointestinal motility thereby slowing the entry of nutrients into the circulation and preventing exaggerated blood glucose excursions [2]. This extrapancreatic effect may indicate that GLP-1 is also clinically relevant to adjunctive insulin T1DM therapy [3–6]. It also underscores the importance of elucidating the mechanisms regulating incretin receptor expression in the relevant extrapancreatic target organs such as stomach. Scarce data is available regarding the expression of the GLP-1R in extrapancreatic target organs in diabetic patients and nondiabetic individuals. We recently demonstrated the presence of GLP-1R in normal human stomach mucosa [7]. This data suggests that GLP-1R is directly involved in regulating gastric function. Furthermore, we showed for the first time that patients with long standing T2DM demonstrated decreased expression of their gastric glands GLP-1R [8] indicating that T2DM diabetes affects the expression of extrapancreatic GLP-1R. Similar data in T1DM patients has not been reported. Thus, it is not known whether T1DM and T2DM differently modulate the expression of GLP-1R in extrapancreatic target organs.

In this work, we examined the expression of the GLP-1R in an incretin target organ, namely, the stomach in chemical and nutrient induced experimental diabetes, in order to elucidate the mechanisms of possible differences in GLP-1R expression in diabetes mellitus with different etiopathogenesis.

## 2. Materials and Methods

### 2.1. Experimental Animals

*Psammomys obesus* (PO) also known as “*Sand rats*” and Sprague Dawely (SPD) outbred laboratory rats were purchased from Harlan Labs Israel (Jerusalem, Israel). PO and SPD were housed under a 12 h light/dark cycle starting at 6 am at constant air temperature 21°C and* ad libitum* access to food and water. All experimental procedures (maintenance, handling, and killing) were approved by the Institutional Animal Care and Use Committee at Assaf Harofe Medical Center and were carried out according to regulations specified in the Israeli prevention of cruelty to animals act.

### 2.2. Experimental Models of HE Diet- and STZ-Induced Diabetes

#### 2.2.1. HE Diet-Induced T2DM

Twelve female PO rats weighting 200.8 ± 9.73 g were used for the study of experimental T2DM. Six animals were fed a commercial high-energy (HE) diet of 3.1 kcal/g for 7 weeks and 6 animals were fed a low-energy (LE) diet of 1.8 Kkal/g for the same time (Koffolk, Israel). In captivity PO maintained on nondiabetogenic LE diet are commonly used as control to PO maintained on diabetogenic HE diet.

#### 2.2.2. STZ-Induced T1DM

Twenty female SPD rats weighing 200–250 g were used for the study of experimental T1DM. STZ (Sigma, St Louis, MI, USA) was dissolved in citrate buffer (pH, 4.5) and injected by a single intraperitoneal injection of 55 mg STZ/kg body weight to 10 of the animals. The remaining SPD rats were injected with saline and served as a control group. Whole blood tail glucose concentrations were measured between 9 am to 11 am, weekly using Free Style Freedom Blood Glucose Monitoring System (Abbott Laboratories, USA). Animals were considered diabetic at blood glucose level ≥ 250 mg/dL. PO and SPD rats were killed 20 and 10 days after development of diabetes, respectively. Following sacrifice, rat abdomens were quickly opened; the stomach was excised along the greater curvature, rinsed in saline, and longitudinally bisected. One-half of the stomach specimen was immediately frozen in liquid N_2_ and kept at −70°C for mRNA extraction and analysis and the other half was immersed in formaldehyde for immunohistochemical analysis.

### 2.3. Real-Time Quantitative (q)PCR Analysis

Samples from upper region of glandular stomach were used for RT (q)PCR assay. Total RNA was extracted using the ZR RNA MicroPrep kit (Zymo Research, Irvine, Ca, USA) and cDNA was synthesized using the Verso cDNA synthesis Kit (ABgene, EPSOM, UK) according to manufacture protocols. Primers for GLP-1R and GAPDH were synthesized by Metabion (Germany) after aligning known sequences of rat and mouse for conserved sequences. PO PCR products were cloned and their sequences were deposited into the NCBI database under the accession numbers FJ834453 and FJ834456, respectively. Primers used for PO and SPD rats are listed in Table 1.

Quantitative Real-Time PCR analysis was performed using a Rotor-Gene (Corbett Life Science, Sydney, Australia) thermocycler and SYBR FAST Universal Master Mix (Kapa Biosystems, Cape Town, South Africa). GLP-1R mRNA levels were normalized to the GAPDH mRNA level and expressed as relative units. In order to exclude amplifying genomic DNA, we picked primers on different exons that had an intron in-between. All products had a single melting peak that was different from that received from the no template control primary primer dimmer.

### 2.4. Immunohistochemical Assays

Stomach samples, fixed in 10% neutral buffered formalin, were routinely processed. Specimens were taken only from the upper region of the glandular stomach adjacent to “the limiting ridge” which is a low fold of tissue separating the glandular stomach from the forestomach. Specimens were paraffin wax embedded and cut into 3-4 *μ*m thick serial sections. Hematoxylin and Eosin (H&E) staining was performed for routine microscopic histopathological examination. Primary monoclonal mouse anti-human whole GLP-1R antibodies (# MAB28141, R&D Systems, Inc., Minneapolis, MN, USA) were used for immunostaining procedure. This monoclonal antibody (mouse IgG_2A_) was produced from a hybridoma resulting from the fusion of a mouse myeloma with B cells obtained from a mouse immunized with NSO cells transfected with whole human GLP-1R (accession number P43220). The IgG fraction of the tissue culture supernatant was purified by protein G affinity chromatography. The specificity of this antibody was verified by its ability to stain GLP-1R-transfected CHO cells but not irrelevant transfectants, that is, positive and negative controls, respectively. Flow cytometry data demonstrate that this anti-GLP-1R antibody specifically recognizes GLP-1R in 96.7% of GLP-1R-transfected BAF/3 cells compared with isotype control antibodies (0.4%) and irrelevant BAF/3CXCR6 transfectants (2.1%) (R&D data, not shown). A concentration range of 3 to 8 *μ*g/mL of these antibodies, together with appropriate secondary reagents (R&D Systems Inc., Minneapolis, MN, USA), was used for immunostaining of paraffin-embedded normal hippocampus human tissue sections (R&D system) and in human stomach mucosa sections of nondiabetic and diabetic patients in our previous works [7, 8].

Immunohistochemical staining of all glandular stomach specimens was performed by Cell and Tissue Staining Mouse Kit HRP-DAB System (# CTS002, R&D Systems, Inc., Minneapolis, MN, USA). Briefly, after deparaffinization and hydration of paraffin-embedded mucosal sections, the specimens were blocked with peroxidase blocking reagent for 10 min, serum blocking reagent for 15 min, avidin blocking reagent for 15 min, and biotin blocking reagent for 15 min. After washing monoclonal primary mouse anti-human GLP-1R antibodies were diluted (3 *μ*g/mL) in Dako antibody diluent (Carpinteria, CA, USA), added to the specimens, and incubated overnight at 4°C. Specimens were then incubated with secondary anti-mouse biotinylated antibodies for 45 min followed by incubation with high sensitivity streptavidin conjugates to horseradish peroxidase (HSS-HRP) for 30 min. Visualization of enzymatic conversion of 3,3′-diaminobenzidine (DAB) chromogen substrate into colored brown precipitate by HRP was performed using a microscope monitor. The stain development was stopped after 4 min. Mayers Haematoxylin (Pioneer Research Chemicals, Colchester, UK) was used for nuclear staining of the samples. GLP-1R immunostained rat pancreatic islets and liver served as positive and negative tissue controls, respectively. Null controls were performed by replacement of GLP-1R primary antibodies with Dako antibody diluent or normal serum during GLP-1R immunostaining of gastric mucosa samples.

### 2.5. Immunohistomorphological Analysis

Light microscopy images (BX-51; Olympus Ltd., Tokyo, Japan) were captured by digital camera Olympus DP72 and merged using Cell Sens Standard 1.5 software (Olympus Ltd., Tokyo, Japan).

#### 2.5.1. Semiquantitative Immunohistomorphological Microscopic Analysis

Semiquantitative immunohistomorphological microscopic analysis was performed by estimation intensity of all GLP-1R immunostained glandular cells in each animal specimen by counting minimum 100 immunostained cells separately in each area (neck, mid, and bottom) of gastric glands. Level of GLP-1 immunostaining intensity was estimated by a progressive scale: 0 = negative staining (background level), 1 = mild staining, 2 = moderate staining, 3 = marked staining, and 4 = strong staining. Results were expressed as mean GLP-1R immunostaining intensity of glandular cells in each gastric gland's area.

#### 2.5.2. Quantitative Immunohistomorphological Microscopic Analysis

Quantitative immunohistomorphological microscopic analysis was performed by counting the GLP-1R immunoreactive and nonimmunoreactive glandular cells using Mac Biophotonic ImageJ (a Java program inspired by National Institute of Mental Health, Bethesda, Maryland, USA) in a minimum of 10 well-oriented high-quality longitudinal cuts of gastric glands. Frequency of GLP-1R immunoreactive cells was determined separately in neck, mid, and bottom gland areas. Total counted glandular cells (mean per animal 425 ± 24.21) were 12760. Results were expressed as a mean percent of GLP-1R immunoreactive cells of total glandular cells number in each gland's area.

The semiquantitative and quantitative immunohistomorphological microscopic analyses were performed by the same investigator in a blind manner.

### 2.6. Statistical Analysis

Data are expressed as mean ± SD. Student *t-test* (two tailed) was used to evaluate the statistical significance of differences between groups. A *P* value of ≤0.05 was considered significant.

## 3. Results

### 3.1. Blood Glucose Levels during HE Diet- and STZ-Induced Diabetes

**Figure 1:**
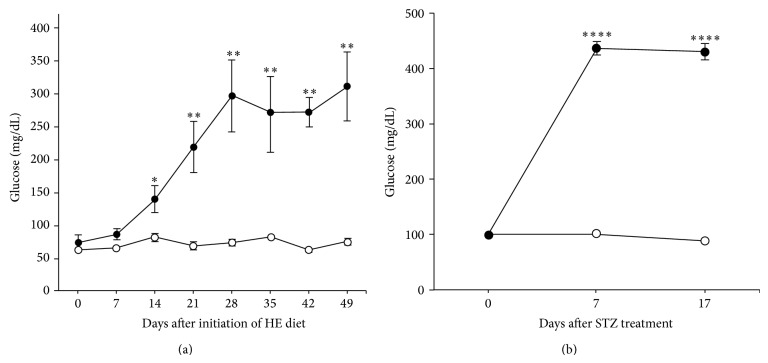
Blood glucose levels in PO and SPD rats after diabetes induction. (a) Gradual increase of blood glucose concentrations after induction of type 2 DM in PO rats by HE diet (full circles). Mild hyperglycemia of approximately 300 mg/dL was observed after 4 weeks and remained stable thereafter as opposed to normoglycemic control animals (empty circles) (^∗^ *P* = 0.01, ^∗∗^ *P* < 0.01). (b) Severe stable hyperglycemia around 450 mg/dL was established one week after single STZ treatment of SPD rats (full circles) as opposed to untreated animals (empty circles) (^∗∗∗∗^ *P* = 0.00001). Data are presented as mean ± SD.

Figure 1 demonstrates blood glucose dynamic in PO rats with/without HE diet and SPD rats with/without single treatment with STZ. Hyperglycemia was detectable in PO after two weeks of HE feeding compared with controls (*P* = 0.03) and gradually increased reaching a plateau at 4 weeks resulting in a stable mild hyperglycemia (approximately 300 mg/dL) compared with LE fed PO (*P* = 0.01) (Figure 1(a)). In SPD rats the stable severe hyperglycemia (greater than 400 mg/dL) was established at one week after single treatment with STZ compared with controls (*P* = 0.00001) (Figure 1(b)). Thus, hyperglycemia had developed faster and was more severe in SPD rats with STZ-induced T1DM compared with PO rats with HE diet-induced T2DM.

### 3.2. HE Diet- and STZ-Induced Diabetes Differently Modulate GLP-1R mRNA Expression in Glandular Stomach of Experimental Diabetes

**Figure 2:**
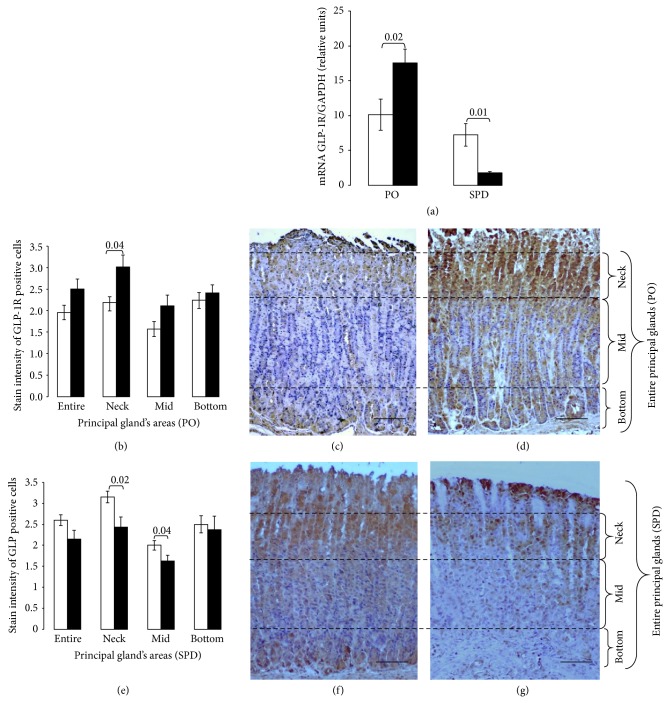
Opposite expression of GLP-1R in glandular stomach of PO and SPD rats after diabetes induction. (a) RT (q)PCR analysis demonstrates that GLP-1R mRNA levels were significantly increased (*P* = 0.02) or decreased (*P* = 0.01) in diabetic PO and SPD rats (black columns), respectively, as compared to normoglycemic untreated animals (white columns). (b, c, d) Semiquantitative immunohistomorphological analysis of glandular GLP-1R expression of diabetic and control PO rats. (b) Significantly increased stain intensity of GLP-1R immunoreactive cells in neck area of principal glands of diabetic (black columns) compared with control (white columns) PO rats. The arbitrary lines presented in illustrations (c) and (d) and (f) and (g) divide the entire gastric glands into three areas named neck, mid, and bottom areas. The neck area includes very narrow isthmus and neck areas of gastric glands; mid area, the upper area of gastric gland base; bottom area, the deep area of gastric gland base. (c, d) GLP-1R immunostained longitudinal sections of glandular stomach mucosa (magnification ×10) demonstrate general increase of stain intensity of GLP-1R immunoreactive glandular cells (brown color) most prominently in the neck of principal glands of diabetic (d) compared with control (c) PO rats. (e, f, g) Semiquantitative immunohistomorphological analysis of glandular GLP-1R expression in diabetic and control SPD rats. (e) Significantly decreased stain intensity of GLP-1R immunoreactive cells in neck (*P* = 0.02) and mid areas (*P* = 0.04) of principal glands of diabetic (black columns) compared with control (white columns) SPD rats. (f, g) GLP-1R immunostained longitudinal sections of glandular stomach mucosa (magnification ×10) demonstrate a general decrease stain intensity (most prominent in the neck area) of GLP-1R immunoreactive glandular cells (brown color) in diabetic (g) compared with control (f) SPD rats. Data are presented as mean ± SD. Bar = 100 *μ*m.

GLP-IR mRNA in control animals (LE-dieted PO and untreated SPD rats) was similar when compared with their respective housekeeping gene (*P* = 0.3) (Figure 2(a)). HE fed diabetic PO demonstrated significant increase of GLP-1R mRNA in glandular stomach compared with control animals (*P* = 0.02). In contrast, STZ treated diabetic SPD rats displayed a significant decrease of GLP-1R mRNA expression as compared with control animals (*P* = 0.01). Thus, HE diet-induced T2DM and STZ-induced T1DM resulted in different modulation of GLP-1R mRNA expression in the glandular stomach.

### 3.3. Histomorphological Features of Glandular Stomach Mucosa in Control and Diabetic PO and SPD Rats

Morphological and morphometric analysis of H&E stained sections demonstrated no differences in gastric glandular mucosa thickness between control LE-diet and HE-diet diabetic PO rats (652 ± 119.2 *μ*m and 672 ± 98.8 *μ*m, resp., *P* = 0.76) as well as in control and STZ treated diabetic SPD rats (615 ± 78.5 *μ*m and 593 ± 87.6 *μ*m, resp., *P* = 0.57). In addition, no desquamation of the surface mucosal epithelium accompanied by diffuse hemorrhage or erosions was detected in diabetic PO and SPD rats. These data demonstrate that HE diet- and STZ-induced diabetes did not alter stomach histomorphology allowing comparative analysis of GLP-1R expression.

### 3.4. GLP-1R Cellular Association and Glandular Distribution in Control PO and SPD Rats

**Figure 3:**
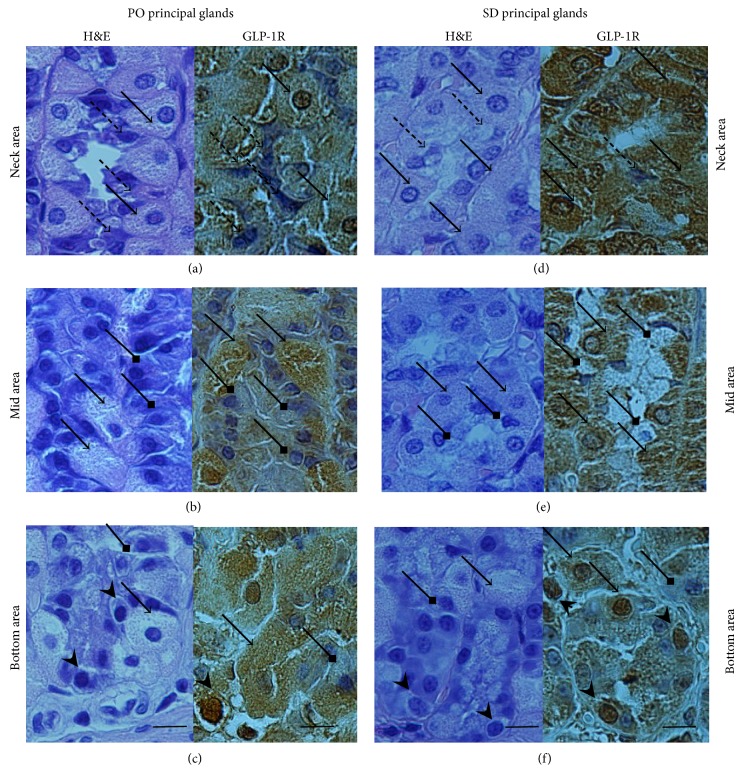
GLP-1R cellular association in gastric principal glands of PO and SPD rats. Longitudinal sections of gastric principal glands of PO (a, b, c) and SD rats (d, e, f). H&E staining (left panels) and GLP-1R immunostaining (right panels) are shown (magnification ×40). (a, d) Neck area of principal glands of PO (a) and SPD (d) rats is dominated by acid secreting parietal cells (solid arrows) which display strong GLP-1R immunoreactivity. Mucous secreting neck cells (broken arrows) demonstrate no GLP-1R immunoreactivity. (b, e) Mid area of principal glands of PO (b) and SPD (e) rats is populated by acid secreting GLP-1R immunostained parietal cells (solid arrows) and chief cells without GLP-1R immunoreactivity (square head arrows). (c, f) Bottom area of principal glands of PO (c) and SPD (f) rats. GLP-1R immunopositive enteroendocrine-like cells (head only arrows), parietal cells (solid arrows), and chief cells (square head arrows) without GLP-1R immunoreactivity are shown. Bar = 20 *μ*m.

**Figure 4:**
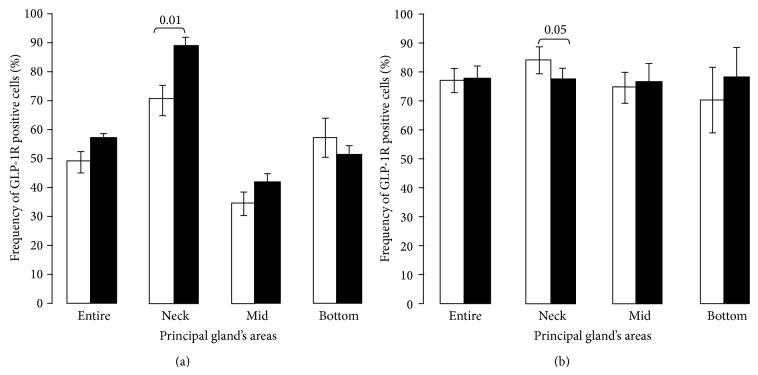
Different frequency of GLP-1R immunoreactive glandular cells in principal glands of diabetic PO and SPD rats. Frequency of GLP-1R immunoreactive cells was determined by quantitative immunohistomorphological analysis in neck, mid, and bottom areas of principal glands. (a) A significant increase of GLP-1R bearing parietal cells in the neck area of principal glands in diabetic PO (black bars) as compared to control animals (white bars) (*P* = 0.01). (b) A significant decrease in GLP-1R bearing parietal cells in diabetic SPD compared with control animals (*P* = 0.05). Data are presented as mean ± SD.

Qualitative histomorphological analysis of H&E stained and GLP-1R immunostained gastric mucosa sections was initially performed to identify specific cell types bearing GLP-1R and its distribution within principal glands (neck, mid, and bottom areas) of control (LE-dieted PO and untreated SPD) rats.  Figure 3 shows that both parietal cells and enteroendocrine like glandular cells displayed strong GLP-1R immunoreactivity. In contrast, neck cells and chief cells demonstrated no GLP-1R immunoreactivity in both rat subgroups. However, overall expression and regional distribution of GLP-1R bearing cells within principal glands were not entirely similar in PO and SPD rats. Both PO and SPD rats demonstrated a predominance of GLP-1R positive parietal cells in the neck area of principal glands (Figures 2(c), 2(f), 3(a), and 3(d)), but these cells were more numerous in all glandular areas in SPD rats (Figures 2(f), 3(e), and 3(f)) as compared with PO rats (Figures 2(c), 3(b), and 3(c)) amounting to 76.5 ± 4.10% and 49 ± 8.10% of entire glandular cells, respectively (*P* = 0.0002) (Figures 4(a) and 4(b)). Thus PO and SPD express GLP-1R in the same glandular cell types but display different distribution and overall expression and of the cells within the gastric glands.

### 3.5. Opposite Effects of HE Diet- and STZ-Induced Diabetes on Glandular GLP-1R Expression

To estimate impact of HE-diet and STZ-induced diabetes on gastric glands GLP-1R expression we performed semiquantitative and quantitative microscopic analysis of GLP-1R immunoreactive glandular cells.

#### 3.5.1. Semiquantitative Immunohistochemical Analysis

Mean stain intensity of GLP-1R immunoreactive glandular cells did not differ significantly in control PO and SPD rats (2.0 ± 0.17 and 2.6 ± 0.13, resp.). However, diabetic PO rats demonstrated an increased GLP-1R immunostaining as compared to control PO (Figures 2(c) and 2(d)). This increase was more prominent reaching a statistical significance in the neck area of the principal glands (*P* = 0.04) (Figure 2(b)). In contrast, STZ treated diabetic SPD rats demonstrated a decreased GLP-1R staining intensity of their glandular cells, which was most prominent in the neck and mid areas of the principal glands compared with control SPD (*P* = 0.02 and *P* = 0.04, resp.) (Figures 3(e), 3(f), and 3(g)).

#### 3.5.2. Quantitative Immunohistomorphological Analysis

HE diet-induced diabetes resulted in significantly increased frequency of GLP-1R immunoreactive cells in neck area of the glands in PO compared with normoglycemic control PO (*P* < 0.01, Figure 4(a)). In contrast, SPD rats with STZ-induced diabetes demonstrated a significantly decreased number of their glandular GLP-1R bearing cells in the same area compared with normoglycemic nondiabetic controls (*P* < 0.05) (Figure 4(b)). Thus, HE diet- and STZ-induced diabetes result in distinct and opposite alterations of GLP-1R expression in the principal gastric glands. Both staining intensity and frequency of GLP-1R bearing cells are increased or decreased in PO and SPD rats, respectively. These changes are most prominent in the neck area of principal glands.

## 4. Discussion

This study is the first to examine the effect of different types of experimental diabetes on GLP-1R expression in an extrapancreatic target organ of the enteropancreatic axis, namely, the glandular stomach. Nutritionally and chemical induced diabetes were chosen in order to elucidate the mechanism(s) associated with GLP-1R expression in T1DM and T2DM. Molecular and immunohistomorphological analysis demonstrated that expression of GLP-1R mRNA encoding protein and its cellular association were similar in nondiabetic PO and SPD rats which are different rodent subgroups. However, both molecular and morphological immunohistochemical analysis revealed that GLP-1R expression was increased in PO rats with HE diet-induced T2DM but decreased in SPD rats with STZ-induced T1DM. The most prominent changes in GLP-1R expression took place in the neck area of the principal gastric glands which is densely populated by acid secreting parietal cells. Taken together, these data show that experimentally induced T1DM and T2DM differently modulate GLP-1R expression in gastric glands.

STZ treated SPD rats with T1DM exhibit insulinopenia, rapid body mass loss [10], and acute severe hyperglycemia along with reduced gastric GLP-1R expression. Several mechanisms may explain the reduced expression of gastric GLP-1R in these animals. In this model of diabetes, pancreatic and extrapancreatic organs are exposed to both direct toxic effect of STZ and to severe hyperglycemia following almost total loss of insulin producing beta cells [11]. It was demonstrated that hyperglycemia itself contributes to downregulation of GLP-1R expression in islets of rat pancreas* in vivo* and* in vitro* [9] and this effect is more prominent after long-term (4 weeks) hyperglycemia* in vivo* [12]. It is also known that diabetogenic dose of STZ not only induces death of pancreatic beta cells [13], but also directly damages gastric mucosa [14]. Moreover, the severity of early STZ mediated gastric mucosal lesions may be further aggravated by long-term diabetic state [14]. In STZ treated diabetic SPD rats, we found decreased extrapancreatic expression of these receptors and reduced frequency of GLP-1R bearing cells in acid producing glands area. At the same time we did not find any histological evidence of gastric mucosal lesions such as desquamation of the surface epithelium with diffuse hemorrhage or erosions. Therefore, it is possible that the GLP-1R reduced expression precedes the pathomorphological changes and may serve as additional marker of early extrapancreatic cellular damage induced by STZ diabetes. Taken together, our data may suggest that combined early STZ and late hyperglycemic mediated cytotoxicity reduce the expression of GLP-1R in gastric glands of rats with STZ-induced diabetes. In addition, we demonstrated a significant loss of parietal cells in the STZ diabetic rats. This may result in decreased gastric acid secretion aggravated by chronic hyperglycemia which has been previously shown to inhibit gastric acid output [15]. This notion is in agreement with the data demonstrating a decreased basal and stimulated acid secretion in rats with long-term STZ-induced diabetes [16]. It should be noted that our study focused on short-term STZ mediated diabetes in which acid secretion remains intact [17]. In addition, GLP-1 is known to be a powerful inhibitor of gastric acid secretion [18]; the downregulation of GLP-1R observed in gastric glands of STZ rats may increase acid secretion in their remaining parietal cells in order to normalize overall acid secretion. The decreased GLP-1R signaling may modify acid secretion directly in parietal cells and/or via paracrine regulation of the cells by somatostatin- and gastrin-secreting enteroendocrine cells [19, 20]. Therefore, it is possible that acid secretion in short-term STZ-induced diabetes remains initially intact due to adaptive response mediated by GLP-1 acid regulation. However, gastric mucosal lesions aggravated by chronic severe hyperglycemia [16] and possible autonomic neuropathy may independently result in irreversible severe acid secretion dysfunction in long-term STZ-induced diabetes. Thus, the exact role of gastric stomach GLP-1/GLP-1R system in regulation of acid secretion in different stage of T1DM remains to be elucidated.

In contrast to STZ diabetes, nutritionally induced diabetes in PO is characterized by innate insulin resistance, beta-cell dysfunction [21], hyperinsulinemia [22], gradual development of mild hyperglycemia, overweight, and upregulated pancreatic and circulated GLP-1 secretion [23]. Increased pancreatic GLP-1R expression and circulating GLP-1 levels were also found in insulin-resistant mice with high-energy diet [24] suggesting adaptive response of pancreatic paracrine and endocrine GLP-1/GLP-1R system in insulin-resistant animals with type 2 diabetes. In our study we found that gastric GLP-1R expression is also enhanced in diabetic PO rats. Thus, upregulation of extrapancreatic GLP-1R and not only its ligand may be an inherent part of the adaptive response to HE diet and development of T2DM in insulin-resistant animals. We found that the frequency of acid secreting parietal cells is increased in diabetic PO rats with relatively mild hyperglycemia. This increase which is probably mediated by increased nutrition may result in increased acid production. In the same time, GLP-1R expression in acid secreting parietal cells and acid regulating enteroendocrine cells was also increased. It is conceivable, therefore, that the increased GLP-1R expression and possible signaling may represent an adaptive inhibition of enhanced gastric acid secretion in response to HE diet. Indeed, gastric acid inhibition in PO diabetic by proton pump inhibitor is associated with improvement of glycaemic control [25]. The increased gastric GLP-1R expression in diabetic PO is in difference to our reported decrease in patients with long-term T2DM [8]. This discrepancy may result from multiple factors including genetic background, nutritional and metabolic differences, drug treatment, disease duration, and possible gastric pathology common to patients with long standing T2DM.

**Figure 5:**
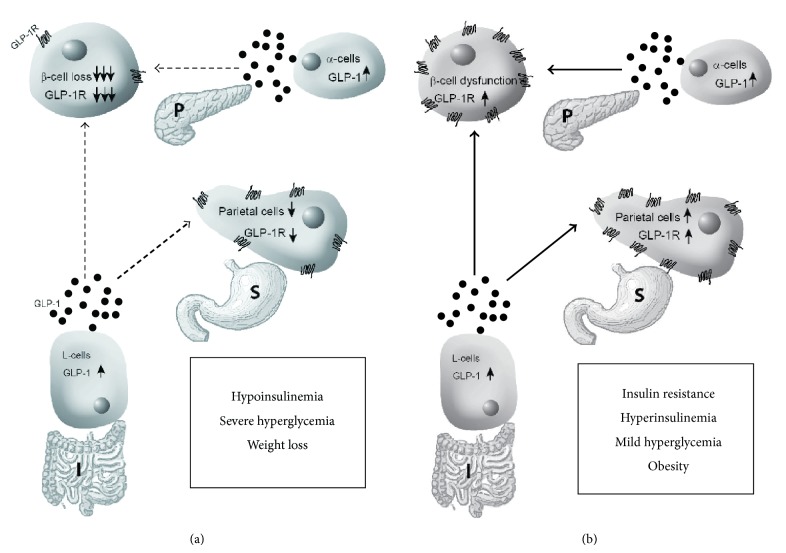
A schematic model displaying the opposite alterations of GLP-1/GLP-1R system in enteropancreatic axis in T1DM and T2DM experimental diabetes. (a) In STZ-induced T1DM, GLP-1 secretion from intestinal L cells and pancreatic alpha cells is increased (upward thin arrows). However, STZ toxicity causes severe loss of pancreatic beta cells and mild loss of extrapancreatic gastric glands parietal cells with the resulting decrease of the GLP-1 receptor (downward thin arrows). The discrepancy between increased GLP-1 secretion and reduced GLP-1R expression results in severely impaired GLP-1/GLP-1R signaling in pancreas and to a lesser degree in the stomach (broken thin and thick arrows). (b) HE diet-induced T2DM is characterized by increased GLP-1R on beta cells in pancreas and gastric parietal cells which are also increased in number as well as increased GLP-1 secretion from pancreatic alpha cells and extrapancreatic intestinal L cells (upward thin arrows). The joint increase in GLP-1 secretion and GLP-1R expression in enteropancreatic axis results in upregulation of endocrine and paracrine GLP-1/GLP-1R signaling in pancreas and stomach (thick arrows). P: pancreas; S: stomach; I: ileum.

Interestingly, endogenous GLP-1 secretion is increased in both models of experimental diabetes [23, 26–28]. However, following STZ administration, although endogenous GLP-1 secretion from the ileum and pancreas is increased, in same time, GLP-1R expression is drastically decreased in pancreatic beta cells and diminished in gastric parietal cell due to direct STZ toxicity augmented by the associated severe hyperglycemia. The disproportion between the increased endogenous GLP-1 secretion and decreased GLP-1R expression in enteropancreatic axis target organs results in an impaired/diminished GLP-1R signaling in pancreas and stomach, respectively (Figure 5(a)). In difference, HE dieted PO diabetic rats demonstrate increased ileal and pancreatic endogenous GLP-1 secretion associated with increased GLP-1R expression [23]. This results in an overactivation of GLP-1R signaling in stomach and pancreas (Figure 5(b)). This effect may be curbed by insulin resistance which has been associated with impaired GLP-1 secretion and beta cell dysfunction [29, 30].

Thus, our data suggest different modulation of GLP-1/GLP-1R system signaling and function in experimental T1DM and T2DM. This may underlie the different clinical efficacy of GLP-1 replacement therapy in patients with T1DM and T2DM [31–33]. GLP-1 replacement therapy has also been advocated in T1DM by several researchers as an adjunctive therapy to insulin. This approach is based on the known extrapancreatic effects of GLP-1 which are assumed to be therapeutically significant even in the absence of endogenous insulin secretion [3–6, 34]. However, this assumption is not based on solid experimental data inasmuch as the expression of GLP-1R in extrapancreatic target organs has not been determined in patients with T1DM. Our data in experimental T1DM demonstrating reduced gastric GLP-1R expression does not support this approach. Alternatively, it is possible that GLP-1 adjunctive therapy in T1DM patients exerts its beneficial effects directly via extrapancreatic target organs other than the stomach or indirectly via the central nervous system [35].

## 5. Conclusion

Chemical and nutrient induced experimental T1DM and T2DM display opposite effects regarding GLP-1R expression in principal gastric glands. This suggests that induced T1DM and T2DM may differently modulate GLP-1R system in extrapancreatic target organs such as the stomach. If extrapolated to other target organs of the enteropancreatic axis it may provide additional support for GLP-1 replacement therapy in T2DM and explain why it remains less effective in T1DM.

## Figures and Tables

**Table 1:** 

Gene name	Forward sequence	Reverse sequence
GLP-1R [9]	GGGTCTCTGGCTACATAAGGACAAC	AAGGATGGCTGAAGCGATGAC
GAPDH	CAGGAGCGAGATCCCGC	CCTTTTGGCCCCACCCT
